# Tracking sexual dimorphism of facial width-to-height ratio across the lifespan: implications for perceived aggressiveness

**DOI:** 10.1098/rsos.211500

**Published:** 2022-05-04

**Authors:** Stephanie Summersby, Bonnie Harris, Thomas F. Denson, David White

**Affiliations:** School of Psychology, University of New South Wales, Sydney, New South Wales 2052, Australia

**Keywords:** aggression, impression formation, face perception, person perception, social cognition, human evolution

## Abstract

The facial width-to-height ratio (FWHR) influences social judgements like perceived aggression. This may be because FWHR is a sexually dimorphic feature, with males having higher FWHR than females. However, evidence for sexual dimorphism is mixed, little is known about how it varies with age, and the relationship between sexual dimorphism and perceived aggressiveness is unclear. We addressed these gaps by measuring FWHR of 17 607 passport images of male and female faces across the lifespan. We found larger FWHR in males only in young adulthood, aligning with the stage most commonly associated with mate selection and intrasexual competition. However, the direction of dimorphism was reversed after 48 years of age, with females recording larger FWHRs than males. We then examined how natural variation in FWHR affected perceived aggressiveness. The relationship between FWHR and perceived aggressiveness was strongest for males at 27–33 and females at 34–61. Raters were most sensitive to differences in FWHR for young adult male faces, pointing to enhanced sensitivity to FWHR as a cue to aggressiveness. This may reflect a common mechanism for evaluating male aggressiveness from variability in structural (FWHR) and malleable (emotional expression) aspects of the face.

## 1. Tracking sexual dimorphism of facial width-to-height ratio across the lifespan: implications for perceived aggressiveness

Humans extract a great deal of socially relevant information from people's faces and make social inferences about unfamiliar people after a single glance. Facial width-to-height ratio (FWHR) is a cue that has been linked to a variety of social inferences and can be calculated as the width of a face divided by the vertical distance between the highest point of the upper lip and the highest point of the eyelids [1–4]. FWHR in men positively correlates with a broad range of social perceptions indicative of formidability such as dominance, aggression, threat and masculinity [5]; although see Dixson *et al*. [6] where researchers found no effects of male FWHR on judgements of masculinity and dominance, and Whitehouse *et al*. [7], who found that prenatal testosterone concentrations positively correlated with men's facial masculinity, but not the FWHR. There is some truth to these perceptions with studies showing that men with relatively larger FWHRs have larger biceps [8], are better physical fighters [9], and report greater dominance and aggressive behaviour [5,10] including domestic violence [11].

The association between FWHR, masculinity and perceptions of aggressiveness has led to the speculation that FWHR is a secondary sexual characteristic—a trait that has been shaped by sexual selection and/or intrasexual competition [12–14]. A key criterion of secondary sexual characteristics is that they are sexually dimorphic during periods of life that are associated with mate selection [15]. However, evidence for sexual dimorphism of FWHR is mixed. A recent meta-analysis found that men had significantly larger FWHRs than women [5] but the effect size was very small (*d* = 0.11) and there are a number of reports of no dimorphism (e.g. [3,14,16,17]). One possible reason for this inconsistency in the literature is that most research on the FWHR has examined a circumscribed range of ages, typically focussing on young adults, where effects of mate selection would presumably be strongest. The FWHR may change across time and the nature of change may differ for males and females, potentially accounting for the observed inconsistency in sexual dimorphism.

Few studies examined sexual dimorphism of the FWHR in age groups other than young people. For instance, Robertson & Kingsley [18] used a sample of Black faces across four age groups (i.e. 20's, 30's, 40's and 50's). Men and women in their 20's and 30's had equivalent FWHRs; however, women in their 40's and 50's had larger FWHRs than men. A study by the same group found no sexual dimorphism across the four decades in FWHR in a sample of White faces [19]. Other research found no evidence for sexual dimorphism among a cohort of elderly White individuals [3]. Another study manipulated the FWHR with computer-generated White male faces intended to be young adults, middle-aged or elderly [20]. Participants rated each face on aggression, wisdom and warmth. The FWHR was positively correlated with perceived aggression in all age groups. The authors also found that a stimulus set of real male faces showed a decline in the FWHR across the lifespan [20], but another study found a small positive correlation between the FWHR and age in men and women [21].

In summary, current evidence is equivocal as to whether there is sexual dimorphism of the FWHR across different ages, and not enough is known about how these changes in the FWHR might influence perceptions of aggression. This may be owing to the tendency for relatively small samples of faces used in individual studies (e.g. [10,22]) or not sampling faces uniformly across the lifespan in the general population (e.g. [1,8,21]). There are also differences in the way that face images are captured, ranging from relatively controlled studio capture (e.g. [3]); to less-controlled images (e.g. [23]); to images taken from social media with unconstrained environmental, camera and capture settings (e.g. [21]).

Evolutionary theory is often evoked to explain sexual dimorphism in the FWHR (e.g. [24]). Sexual selection, in particular is thought to have influenced the evolution of the FWHR. Sexually selected traits such as the FWHR can serve as cues to dominance and formidability towards same-sex rivals or used to attract potential opposite-sex mates. The latter notion is unlikely to be true as larger FWHRs in men are relatively unattractive to women [5], although one study found that men with relatively large FWHRs were viewed as attractive to women for a short-term relationship [25]. Nonetheless, men with relatively larger FWHRs have more offspring, and stronger sexual motivation than men with lower FWHRs, which suggests a relationship between FWHR and fitness [26]. Research has also found that higher FWHR is associated with greater sexual desire in men [27], but not in women [27,28].

Stronger evidence supports the notion that the FWHR may have been a sexually selected trait in men, which signals formidability to other male rivals [29]. Similarly, men with relatively larger FWHRs have the ability to outcompete men with lower FWHRs in sports [30,31], business [32,33], physical fights [9] and warfare [26], all of which may lead to status, wealth and obtaining other reproductively relevant resources and hence greater mating opportunities.

The evolutionary theorizing regarding sexual selection thus far has been limited to men and is agnostic regarding age-related changes. The evolutionary prediction for men seems relatively clear. Other than during childhood when the FWHR is highest, the FWHR should become largest in men during the highest period of intrasexual competition and physical strength (i.e. 18–30 years old). After that, the FWHR should remain relatively stable or decline in men. In terms of perceptions of aggressiveness across time and gender, it is likely that the FWHR should show the strongest correlations with perceived aggressiveness during the same period of high intrasexual competition in men.

For women, there is no clear prediction for the sizes of the FWHR across the lifespan or relationship with perceived aggressiveness as the FWHR may be a sexually selected trait in men, but not women. Studies of male and female faces showed a slow decline in FWHR from the age of 20 until the age of 40 for both men and women [13]. The authors attributed this result to the lengthening of the face with age. Although the authors did not examine age effects directly, Durkee & Ayers [34] speculated that the age-related decline in the FWHR may cause perceivers to view older people as less threatening than younger people.

**Figure 1.**
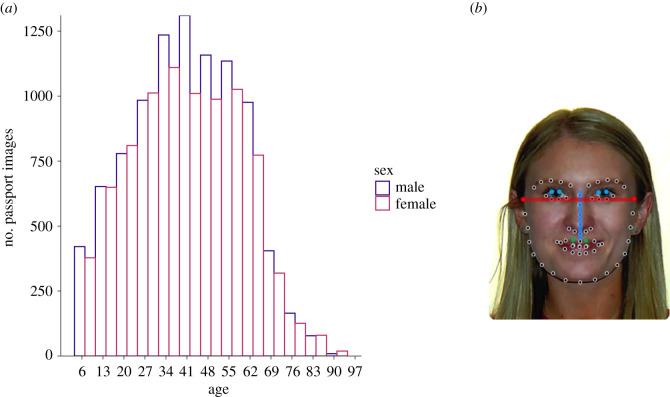
(*a*) Histogram of the age distribution of the 17 607 face images in the passport dataset. (*b*) Sixty-eight facial landmark points were detected by the facial landmark detection algorithm, and the points were used to calculate facial width (red) and height (blue and green). To protect the identity of the people contributing their passport images for use in the study, we were unable to publish individual images that were actually used in the study. The passport photograph used here of the lead author is used for illustration only.

Here we sought to address some of the inconsistencies in the knowledge base surrounding the relationships between the FWHR, sex, age and perceived aggressiveness. We used a unique face image database of passport images of over 17 607 Australian citizens who consented for their face to be used for research when applying for their passport. In addition to being very large, this dataset had four properties that enabled a systematic examination of faces across the lifespan. First, images were relatively controlled in terms of head angle, pose and camera-to-subject distance, thereby minimizing confounding effects of imaging conditions on face shape. Second, because 57% of Australians have passports [35], it was a relatively broad sampling of faces from the general population. Third, each face was coded with reliable age information verified via official records at the time of application. Fourth, the database represented a relatively broad sampling of facial age (figure 1*a*).

To enable efficient analysis of this large dataset, we used an automatic face landmark detection algorithm to compute the FWHR [36]. We then recruited 121 participants to rate the perceived aggression of males and females with high and low FWHR, to examine the strength of the relationship between the FWHR and perceived aggression across the lifespan. To pre-empt our results, we show that FWHR is larger for males only in a narrow age band in early adulthood, aligning with the period of life most commonly associated with mate selection and intrasexual competition, with larger FWHR in females found in mid- to late-adulthood. Aggression ratings to male faces appear to be most sensitive to differences in FWHR during this period, pointing to an increased perceptual sensitivity to FWHR in young adult males.

## 2. Methods

### 2.1. Passport image database

The main analysis described in this paper is based on a database of 17 607 passport images of 9307 male and 8300 female Australian citizens, who consented for their photographs and associated sex and age information to be used in research when applying for a passport. The age distributions of male and female passport application photos are shown in figure 1*a* (*M* = 41.3, s.d. = 17.9, range: 6–93 years). The sample contained individuals from a range of different ethnicities and conformed broadly to the demographics of Australian citizens.

For a subset of individuals in the passport database, we also had an image of their face from a previous passport application, enabling us to examine the stability of the FWHR in individuals over time. This subset contained images of 259 female and 251 male Australian citizens (*n* = 510) with an average of 11.21 years (s.d. = 1.42 years) between older passport images and the most recent passport image. For the older passport images in this subset, the average age at issue was 32.9 years (s.d. = 9.4 years) and for the more recent images, it was 44.1 years (s.d. = 9.3 years).

### 2.2. Facial landmark detection and automatic facial width-to-height ratio computation

Given the large image dataset used in this study, we calculated FWHR using an automatic face landmark detection algorithm described by Kazemi & Sullivan [36]. The dlib.net implementation we used was trained on the I-BUG 300-W face landmark dataset, which consists of 600 (300 indoor and 300 outdoor) facial images that vary in expression, illumination and pose [37–39]. Key landmarks were extracted from the output of this algorithm to calculate FWHR, as shown in figure 1*b*. These key landmarks were used to estimate the location of the top of the eyelids, top of the lips, and the left and right temples. Consistent with previous work, facial height was calculated as the vertical distance between the lips and eyelid, and width was the horizontal distance between the temples (see [1–4]). FWHR was then calculated for each image by dividing facial width by facial height.

We validated our automated FWHR measurement by comparing its output to manual human measurement using two different datasets. First, we used a database of 2222 face images compiled by Bainbridge *et al*. ([40,41]; the 10 K US Adult Faces Database) that were gathered from images returned by a Google Image searches for various names created by a random name generator. This was a strict test of our automatic approach because images in this dataset are variable in terms of expression, pose and illumination, which produces a more challenging computational problem than when reading facial landmarks from passport photos, which are uniformly looking straight ahead with neutral pose and conform to a series of quality control checks. We also validated our measurement approach on a second set of 100 images sampled at random from the larger passport database that was used in the main analyses reported in this paper. We found high correspondence between manual and automated methods for both datasets, *r*s ≥ 0.79, *p*s < 0.001. Full details of this validation analysis are provided in the electronic supplementary material, ‘Validation of Automatic Facial Landmark Detection Algorithm’.

### 2.3. Aggressiveness ratings

We recruited 121 undergraduate students from the University of New South Wales (UNSW) Sydney to rate the perceived aggressiveness of a subset of images from the passport image database (76 female, average age = 19.64 years, s.d. = 2.92 years, range = 17–37 years). The aim of this study was to examine the effect of FWHR differences in a naturalistic sample of faces on perceived aggression. To do this, we selected a sample of 1893 male and female passport images with extreme high- and low-FWHR values. On each trial, participants were presented with an image of a face and asked to answer the question ‘how aggressive would this person be if provoked?’ using a 7-point scale from 1 (not at all) to 7 (extremely). Full details of the methods used in this study are provided in the electronic supplementary material, ‘Aggressiveness Ratings'.

## 3. Results and discussion

### 3.1. Sexual dimorphism of facial width-to-height ratio across the lifespan

The overall average FWHR of the 17 607 faces used in this analysis was 2.18 (s.d. = 0.18; See ‘Sexual Dimorphism of FWHR Across the Lifespan’ in the electronic supplementary material for the average FWHR for each sex and age group). The effects of gender and age on FWHR were analysed using a 2 (sex: male or female) × 10 (age: 6–12, 13–19, 20–26, 27–33, 34–40, 41–47, 48–54, 55–61, 62–68 or 69+) factorial between-subjects ANOVA. There was a significant main effect of age (*F*_9,17587_ = 88.73, *p* < 0.001, $\eta_{p}^{2}=0.043$, 95% confidence interval (CI) [0.037, 0.049]), with an overall tendency for FWHR to decrease with age. This result supports the previous findings of Hehman *et al*. [20] and Hodges-Simeon *et al*. [13], where the authors found that FWHR decreased with age. The main effect of sex was also significant (*F*_1,17587_ = 17.05, *p* < 0.001, $\eta_{p}^{2}=0.001$, 95% CI [0.0003, 0.002]), with females (*M* = 2.19, s.d. = 0.17) having a larger FWHR in general compared to males (*M* = 2.18, s.d. = 0.18). More importantly for our study, main effects were qualified by a significant interaction between age and sex (*F*_9,17587_ = 12.89, *p* < 0.001, $\eta_{p}^{2}=0.007$, 95% CI [0.003, 0.009]), pointing to different trajectories of FWHR over the lifespan for males and females.

**Figure 2.**
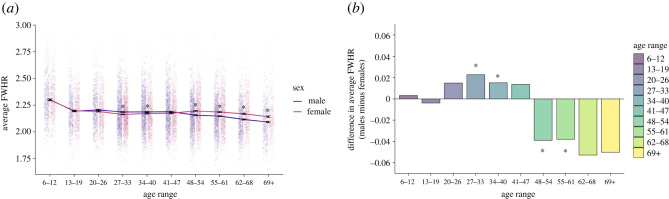
(*a*) FWHR of 17 607 male and female faces by age. Error bars represent standard errors. Jittered data points of the FWHR of each male and female facial image are plotted behind the line graph to represent the data distribution. Four outliers with FWHR > 3 are not displayed. (*b*) Mean difference between the male and female FWHR at each age range, showing higher male FWHR for faces between 27 and 40 years, and higher female FWHR for faces older than 48 years. For both graphs, asterisks represent statistical significance at *p* ≤ 0.05.

As can be seen from figure 2, males had a significantly greater FWHR compared to females at ages 27–33 (*F*_1,17587_ = 11.02, *p* = 0.001, $\eta_{p}^{2}=0.001$) and 34–40 (*F*_1,17587_ = 4.15, *p* = 0.043, $\eta_{p}^{2}<0.001$). In comparison, females had a significantly greater FWHR compared to males at ages 48–54 (*F*_1,17587_ = 27.77, *p* < 0.001, $\eta_{p}^{2}=0.002$), 55–61 (*F*_1,17587_ = 24.96, *p* < 0.001, $\eta_{p}^{2}=0.001$), 62–68 (*F*_1,17587_ = 33.06, *p* < 0.001, $\eta_{p}^{2}=0.002$), and 69 and older (*F*_1,17587_ = 19.10, *p* < 0.001, $\eta_{p}^{2}=0.001$). We therefore observed sexual dimorphism in younger adulthood (27 to 40 years of age; males > females) as well as later in life (48 years of age and older; females > males). Critically however, and somewhat surprisingly, the direction of the dimorphism was reversed in these two age ranges.^1^

### 3.2. Stability of facial width-to-height ratio in individuals over time

**Figure 3.**
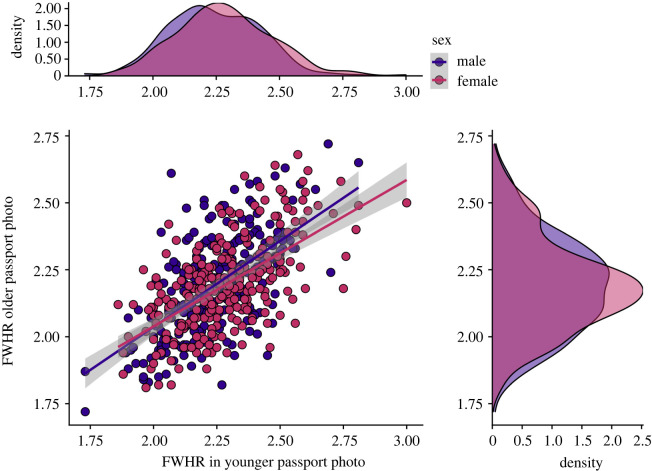
Correlation between individuals' FWHR in their younger and older passport photographs shows stability in FWHR over time.

Because some individuals had more than one passport image in the Australian passport image database, we were able to examine the degree of stability in FWHR as these individuals grew older. Correlation between FWHR between two photographs of the same person taken an average of 11 years apart is shown in figure 3. We found a strong correlation in FWHR between older and younger images for both males (*r* *=* 0.62, *p* < 0.001) and females (*r* *=* 0.61, *p* < 0.001), confirming for the first time, to our knowledge, that this is a relatively stable source of individual difference in facial appearance across the lifespan.

### 3.3. Relationship between perceived aggressiveness and facial width-to-height ratio across the lifespan

**Figure 4.**
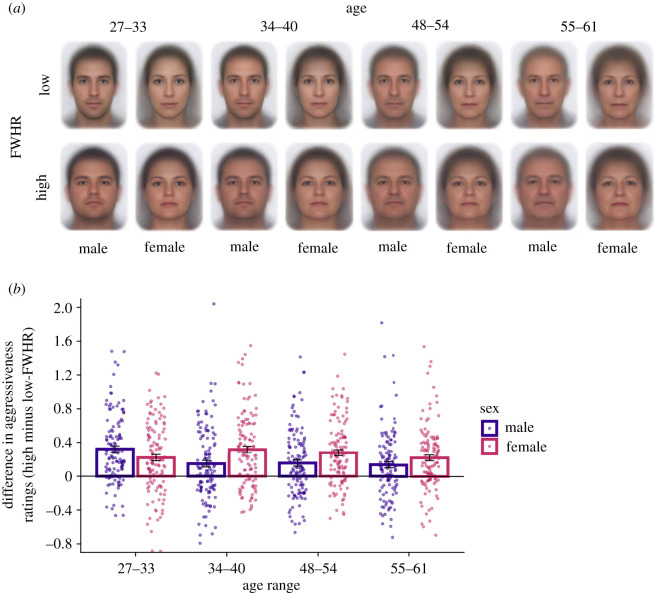
(*a*) Averages of the individual face images used in the perceived aggressiveness rating study. To protect the identity of the people contributing their passport images for use in the study, we were unable to publish individual images and so averages provide a visual representation of experimental stimuli without compromising privacy. Each image is an average of between 114 and 124 images. (*b*) Difference between the average aggressiveness ratings of the high- and low-FWHR faces in each sex and age group, showing higher aggressiveness ratings for high-FWHR faces. Error bars represent standard errors. Jittered data points of the difference score for each individual rater are plotted behind the bar graph to represent the data distribution.

To examine the effect of FWHR differences in a naturalistic sample of faces on perceived aggression, we asked 121 participants to rate perceived aggressiveness of 1893 male and female faces taken from the database of Australian passport photographs. For each age range from 27 to 61 years, we selected faces that had either a high- or low-FWHR relative to other faces in their age range. Mean differences between high- and low-FWHR faces were ≥0.57 for all sex and age groups. The average FWHR of all high and low-FWHR groups were approximately 1 s.d. above and below the mean FWHR, respectively. Averages of the high- and low-FWHR faces shown to participants in the experiment are shown separately by sex and age group in figure 4*a*. We chose to sample images from the age blocks 27–33, 34–40, 48–54 and 55–61, as these blocks displayed significant differences in FWHR between males and females in our analysis of sexual dimorphism of FWHR across the lifespan, with the direction of this difference reversing in younger (27–33, 34–40) versus older faces (48–61). These age blocks therefore provided the most informative comparison for examining how FWHR sexual dimorphism changes in FWHR across ages influence perceptions of aggression.^2^

To aid comprehension of the pattern of results, we calculated difference scores for each participant by subtracting their average aggressiveness rating for high-FWHR faces from their average aggressiveness rating for low-FWHR faces for each sex and age group (for analysis of raw rating data see the electronic supplementary material, ‘Relationship Between Perceived Aggressiveness and FWHR Across the Lifespan’). Positive difference scores indicate that high-FWHR faces had higher perceived aggressiveness than low-FWHR faces. Visual inspection of these scores in figure 4*b* shows the predicted pattern of higher aggressiveness ratings for high-FWHR faces, with positive values for both males and females at all ages. More importantly, the extent to which FWHR modulated aggressiveness ratings also appears to vary by face age, especially for male faces.

We formally analysed the perceived aggressiveness data using a 2 (sex: male or female) × 4 (age: 27–33, 34–40, 48–54 or 55–61) repeated-measures ANOVA. Surprisingly there was a significant main effect of sex (*F*_1,120_ = 6.93, *p* = 0.010, $\eta_{p}^{2}=0.055$), with aggressiveness ratings to females (*M* = 0.26) being modulated by FWHR to a greater extent than for male faces (*M* = 0.19). We also found a significant main effect of age (*F*_3,360_ = 4.53, *p* = 0.004, $\eta_{p}^{2}=0.036$), with difference scores generally decreasing over time. Qualifying these main effects, we found a significant two-way interaction between sex and age (*F*_3,360_ = 8.74, *p* < 0.001, $\eta_{p}^{2}=0.068$). Males had a significantly larger average difference score than females in the 27–33 age range (*M*_diff_ = 0.10, *p* = 0.031), whereas females had a significantly larger average difference score than males in the 34–40, 48–54 and 55–61 age ranges (all *M*_diffs_ ≥ 0.09, all *p*s ≤ 0.018). Additionally, for females, faces at ages 34–40 had a significantly larger average difference score than faces at ages 27–33 and 55–61 (both *M*_diffs_ = 0.10, both *p*s ≤ 0.016). For males, faces at ages 27–33 had a significantly larger average difference score than faces in the other three age ranges (all *M*_diffs_ ≥ 0.17, all *p*s < 0.001). There were no other significant differences between age groups for males or females (all *M*_diffs_ ≤ 0.06 and all *p*s ≥ 0.086), and the effects reported here were not modulated by participant gender (see the electronic supplementary material, ‘Participant Gender and Relationship Between Perceived Aggressiveness and FWHR Across the Lifespan’).

## 4. General discussion

The present study was a large-scale investigation into sexual dimorphism in the FWHR across the lifespan. We found that sexual dimorphism was present in our sample of over 17 000 Australian citizens. In younger to middle adulthood, the FWHR in men was greater than the FWHR in women. From the age of 48 onwards, this pattern was reversed such than women had larger FWHRs than men. One qualification is that the differences between FWHRs in men and women were very small across the age groups. These small effect sizes are consistent with that reported for sexual dimorphism in a meta-analysis (*d* = 0.11; [5]).

The predicted sexual dimorphism was therefore only observed in a surprisingly narrow age band in early adulthood. Yet this pattern in young adults is consistent with the view that FWHR is an evolutionarily important cue to physical formidability, as sexual dimorphism in this age band aligns with the period of life most commonly associated with mate selection and intrasexual competition. The reversal of this dimorphism in middle and late-adulthood, with progressively larger female compared to male FWHR, is more difficult to explain. It is possible that there are broader physical changes in ageing that explain the pattern. For example, because body mass index (BMI) is moderately correlated with the FWHR (*r* = 0.31; [5]), one possibility is that age-related BMI changes are different for males and females. Other possibilities are that the reversal in dimorphism is connected to age-related structural changes to the faces, such as differences in the rate of face lengthening with age [42]. Other possibilities are that increasingly fewer males with higher FWHR apply for passports later in life—perhaps because many men with the largest FWHR may be removed from society via incarceration [43] or early mortality relative to women—or that the difference is affected by changes in head pose behaviour in males and females of different ages [44].

We also tested the relationships between the FWHR and perceived aggressiveness in men and women across the lifespan. In their meta-analysis, Geniole *et al*. [5] found that the relationship between the FWHR and perceived aggressiveness was stronger for younger faces than older faces, but there was no evidence of moderation by sex. By contrast, we found that the relationships between the FWHR and perceived aggressiveness for males was strongest for the youngest age group of faces (27–33 years old), but from 34–61 years old, this relationship was strongest for female faces. These results suggest that the effect of FWHR on perceived aggressiveness ratings varies as a function of age and sex.

Moreover, the effect of FWHR on perceived aggressiveness was somewhat independent from physical variation in FWHR in these age groups. Aggressiveness ratings to faces in the 34 to 40 age range show greater modulation for *female* faces, despite there being more physical FWHR variation in *male* faces (see the electronic supplementary material, figure S4). This shows increased sensitivity to FWHR in the youngest male faces when people evaluate perceived aggressiveness, albeit restricted to a relatively narrow age band. This is consistent with results showing that people are more sensitive to threatening emotional expressions in male compared with female faces [45,46] and may point to a common mechanism responsible for processing FWHR-related and expression-related cues to threat. In face perception research more broadly, it has been proposed that our social impressions of structural aspects of faces are shaped by social learning of facial expressions [47,48], for example, that trustworthiness judgements from structural properties of faces are linked to transient changes such as smiling or warm expressions (e.g. [49]). Future work examining whether other threat cues are also modulated by face age can potentially help to resolve whether similar social learning mechanisms are involved in perceived aggressiveness.^3^

Another potential explanation of these findings is that the apparent increase in sensitivity to FWHR cues in young males was owing to participants being mostly undergraduate students. For face identity processing at least, there is consistent evidence that people develop perceptual expertise specifically for faces fitting the viewer's demographic profile, including faces of the same age as the viewer [51,52]. This raises the possibility that the apparent perceptual sensitivity to FWHR in young faces that we observe may be specific to the younger participants in our study. However, we note that this ‘own age effect’ is reported mostly in identity memory-based recognition tasks and is not consistently found for other types of identity processing task formats [53], or for other types of face judgements [54].^4^ Moreover, participant's age is not known to affect perceptions of aggression [56]. Nevertheless, this is an intriguing question that could be addressed in future work.

The enhanced effect of FWHR on aggressiveness for young men is consistent with evolutionary perspectives on the FWHR as a cue to physical formidability. However, the relationship between the FWHR and aggressiveness for women in middle and late-adulthood is more difficult to explain. Physical variation in FWHR in these age groups was greater for male faces (electronic supplementary material, figure S4), but the effect this variation had on aggressiveness ratings was higher for female faces (figure 4). The differences in ratings of aggression for younger and older men and women may be related to age-specific facial adiposity (the perception of weight in the face). Higher facial adiposity had been associated with higher perceptions of male facial masculinity [57], and lower perceptions of female facial femininity [58]. Thus, one possibility is that age-related changes in facial adiposity are different for males and females, and could be contributing to the sex differences in FWHR and perceived aggressiveness.

Another plausible explanation is that differences in sensitivity to FWHR cues were mediated by widely held stereotypes of masculinity and femininity in younger and older men and women. Men with relatively larger FWHRs are considered masculine, unattractive and physically formidable [5]. As in previous research, we found that these men were also considered likely to become aggressive if provoked [5]. This perception was largest among young men and faded with time. This finding is consistent with the stereotype of older men becoming weaker and less formidable with age, while younger men with relatively large FWHRs were probably viewed as ‘fighting fit’. By contrast, younger women are stereotyped as more feminine, attractive and passive than older women. Thus, participants' judgements of aggressiveness may have been relatively unaffected by the FWHR of the young women. However, stereotypes of older women can be particularly harmful, as they lead to appearance-based discrimination [59]. In this case, the larger FWHRs may have elicited a global negative evaluation bias because the women were not stereotypically attractive, feminine women.

These proposals should be treated as speculative for now. Indeed, there should be some caution when interpreting associations between FWHR and social impressions more generally. The use of discrete anthropometric ratios can sometimes be misleading owing to their associations with other surrounding measures in the body and face (e.g. [60]). This can lead to effects emerging as a consequence of interactions with other interrelated traits (e.g. attractiveness, facial masculinity, facial maturity and anger resemblance, [56]) rather than a consequence of the ratio itself. Therefore, it is important for future research to explore potential traits that may be interacting with the FWHR to impact judgements such as perceived aggressiveness in younger and older low- and high-FWHR faces.

Notwithstanding, to our knowledge our results provide the most comprehensive analysis of FWHR across the lifespan to date. We show that the sexual dimorphism of this trait is consistent with a secondary sexual characteristic that signals formidability in young males. We also show that perceivers were particularly sensitive to FWHR variation in young faces when evaluating perceived aggressiveness. Understanding the causes of face age dependency on perceptions of aggressiveness would be a worthwhile focus of future work.

## Data Availability

The materials and datasets generated and/or analysed for this article are available here: https://osf.io/uw3tk/?view_only=20dad0b2edf4467e8d2a56bc14bfabea. Electronic supplementary material is available online [61].

## Footnotes

1 Face stimuli reflected the ethnic diversity present in Australia. To examine whether face ethnicity affected our results, we repeated this analysis in a subsample of 3225 Caucasian faces. Details of this analysis are provided in the electronic supplementary material ('Sexual Dimorphism of FWHR Across the Lifespan For Caucasian Faces’) and replicate the pattern of results in the main analysis.

2 Here we focused on the relationship between FWHR sexual dimorphism and perceived aggression, but there is also likely to be an association between FWHR and perceived aggressiveness for age ranges where there was no evidence of sexual dimorphism. For example, in the absence of sexual dimorphism, one study of a younger Chinese cohort found that men's FWHR was positively associated with intimate partner violence but not trait dominance. Women's FHWR was positively associated with trait dominance, but not violence [11].

3 Facial hair is also an important age-related sexually dimorphic feature that has been linked to increased perceptions of masculinity dominance and aggressiveness [50]. As such, further research may also wish to explore how facial hair influences the relationship between the FWHR and perceptions of aggression.

4 Additionally, analysis of participant gender is reported in the electronic supplementary material, showing no influence on perceived aggressiveness of male and female faces, despite participant gender showing an interaction with face gender in face recognition memory [55].
